# Istaroxime in Acute Heart Failure and Early Cardiogenic Shock: A Calcium-Cycling Approach to Inotropic Therapy

**DOI:** 10.3390/ijms27135779

**Published:** 2026-06-26

**Authors:** Beata Krasińska, Giuseppe Maria Raffa, Calogera Pisano, Vincenzo Nuzzi, Paolo Manca, Krzysztof J. Filipiak, Mansur Rahnama, Anna Olasińska-Wiśniewska, Mariusz Kowalewski, Zbigniew Krasiński, Piotr Suwalski, Ewelina Grywalska, Tomasz Urbanowicz

**Affiliations:** 1 Department of Hypertensiology, Angiology, and Internal Medicine, Poznan University of Medical Sciences, 1/2 Długa Street, 61-848 Poznań, Poland; 2 Department of Research, IRCCS ISMETT (Mediterranean Institute for Transplantation and Advanced Specialized Therapies), 90127 Palermo, Italy; 3 Department of Precision Medicine in Medical Surgical and Critical Area (Me.Pre.C.C.), University of Palermo, 90134 Palermo, Italy; 4 Department of Clinical Cardiology and Heart Failure, IRCCS ISMETT (Mediterranean Institute for Transplantation and Advanced Specialized Therapies), 90127 Palermo, Italy; 5 The Centre of Postgraduate Medical Education, 99/103 Marymoncka Street, 01-813 Warsaw, Poland; 6 Department of Dental Surgery, Medical University of Lublin, 6 Chodźki Street, 20-093 Lublin, Poland; 7 Cardiac Surgery and Transplantology Department, Poznan University of Medical Sciences, 1/2 Długa Street, 61-848 Poznań, Poland; 8 Department of Cardiac Surgery, Centre of Postgraduate Medical Education, Central Clinical Hospital of the Ministry of Interior, 02-507 Warszawa, Poland; 9 Department of Vascular, Endovascular Surgery, Angiology and Phlebology, Poznan University of Medical Sciences, 1/2 Długa Street, 61-848 Poznań, Poland; 10 Department of Experimental Immunology, Medical University of Lublin, 20-093 Lublin, Poland

**Keywords:** istaroxime, inotropes, AHF, heart failure, cardiogenic shock

## Abstract

Acute heart failure (AHF) and cardiogenic shock (CS) remain major causes of cardiovascular morbidity, mortality, and healthcare utilization worldwide. Although inotropic agents are central to the management of low-output states, their clinical utility is fundamentally constrained by mechanisms that increase myocardial oxygen consumption, disrupt calcium homeostasis, and promote arrhythmogenesis, without improving long-term outcomes. These limitations reflect not only pharmacological shortcomings, but a broader conceptual reliance on amplification of intracellular calcium flux as the primary means of augmenting contractility. While effective in increasing cardiac output, this strategy imposes substantial energetic and electrophysiological costs and fails to address key abnormalities of the failing myocardium, including impaired calcium recirculation and diastolic dysfunction. Istaroxime is a first-in-class agent that combines Na^+^/K^+^-ATPase inhibition with enhancement of sarcoplasmic reticulum Ca^2+^-ATPase (sarcoplasmic reticulum Ca^2+^-ATPase isoform 2a (SERCA2a)) function, thereby modulating both calcium availability and reuptake. This dual mechanism promotes a more coordinated pattern of excitation–contraction coupling, integrating systolic augmentation with improved diastolic relaxation. Early clinical studies demonstrate a distinct hemodynamic profile characterized by increased stroke volume, preservation of heart rate, and stabilization or elevation of arterial pressure. These properties suggest a potential role for istaroxime in specific hemodynamic phenotypes, particularly hypotensive AHF and early cardiogenic shock, where conventional inotropes are limited by tachycardia or vasodilatory effects. However, current evidence is limited to phase II studies focused on hemodynamic endpoints, and the impact of istaroxime on survival, organ function, and disease progression remains unknown. Istaroxime represents a mechanistically distinct approach to inotropic therapy, shifting the paradigm from calcium amplification toward partial restoration of calcium cycling. Its clinical relevance will depend on whether this strategy can translate into improved patient outcomes—an objective that has thus far eluded the entire class of inotropic agents.

## 1. Introduction

Acute heart failure (AHF) and cardiogenic shock (CS) remain among the most challenging syndromes in contemporary cardiovascular medicine, representing the convergence of advanced myocardial dysfunction, systemic hypoperfusion, and multiorgan vulnerability [1,2,3,4]. Despite substantial progress in guideline-directed medical therapy for chronic heart failure, acute decompensation continues to impose a disproportionate burden on healthcare systems, accounting for more than one million hospitalizations annually in both Europe and the United States and remaining the leading cause of hospitalization in individuals older than 65 years [5,6].

The epidemiological trajectory of AHF is particularly concerning. With an aging population, increasing prevalence of multimorbidity, and improved survival from chronic heart failure, the absolute number of patients experiencing acute decompensation is expected to rise steadily over the coming decades. Hospital mortality in unselected AHF populations ranges from 4% to 7%, but this risk increases sharply in the presence of haemodynamic compromise [7,8]. In patients with hypotension or cardiogenic shock, mortality frequently exceeds 30–50%, underscoring the transition from a congestive syndrome to a state of systemic circulatory failure [9,10,11].

Importantly, patients requiring inotropic support represent a distinct biological and clinical phenotype characterized by low cardiac output, impaired end-organ perfusion, and heightened neurohormonal activation [12,13,14]. This subgroup is associated with prolonged intensive care utilization, recurrent hospitalizations, and high resource consumption, reflecting both the severity of disease and the limitations of current therapeutic strategies. The economic burden is further amplified by the need for invasive monitoring, mechanical circulatory support, and management of treatment-related complications, including arrhythmias, hypotension, and renal dysfunction.

Within this context, inotropic agents remain a cornerstone of therapy for low-output states [15,16], yet their role is inherently paradoxical. While capable of rapidly augmenting cardiac output and stabilizing hemodynamics, conventional inotropes have consistently failed to improve long-term outcomes. This limitation is not incidental but mechanistically intrinsic. Most currently available agents—including β-adrenergic agonists and phosphodiesterase-3 inhibitors—achieve their effects by amplifying intracellular calcium flux, a strategy that increases myocardial oxygen consumption, promotes electrical instability, and imposes substantial energetic stress on an already compromised myocardium [17,18,19,20].

The clinical consequences of this paradigm are well documented. Observational registries and randomized trials have repeatedly demonstrated that improvements in hemodynamic parameters do not translate into survival benefit. For example, in the Dobutamine Compared with Milrinone Trial (DOREMI) [21], no significant difference in mortality or major outcomes was observed between dobutamine and milrinone in patients with cardiogenic shock, reinforcing the concept that cAMP-mediated calcium amplification—irrespective of the upstream pathway—fails to alter the disease trajectory. Similarly, trials of calcium sensitizers such as levosimendan (e.g., SURVIVE, REVIVE II) [22,23] have demonstrated hemodynamic and symptomatic improvements without consistent reductions in mortality, highlighting the persistent dissociation between physiological effects and clinical outcomes.

This recurring pattern suggests that the limitations of inotropic therapy extend beyond pharmacology to reflect a deeper conceptual issue: the reliance on increasing calcium availability as the principal means of augmenting contractility. In the failing myocardium, however, the dominant abnormality is not simply insufficient calcium, but impaired calcium cycling—characterized by reduced sarcoplasmic reticulum reuptake, diastolic calcium accumulation, and inefficient excitation–contraction coupling [24,25]. Amplifying calcium flux in this context may transiently improve systolic performance but does so at the cost of increased energetic demand and arrhythmogenic risk, without correcting the underlying defect.

**Figure 1:**
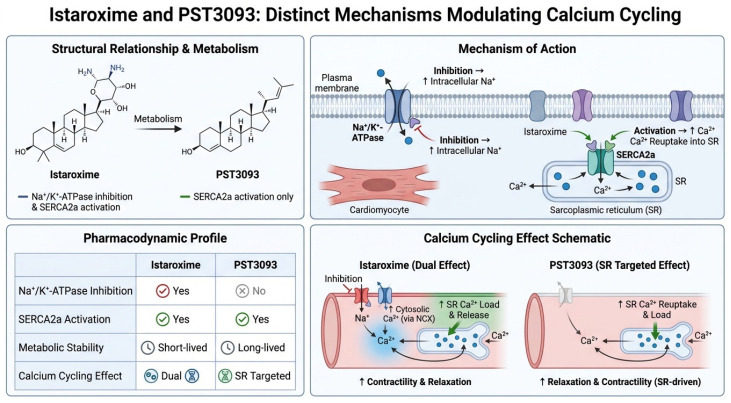
Chemical structure of istaroxime and major metabolite PST3093 (istaroxime metabolite). Istaroxime is a synthetic androstanedione derivative that combines inhibition of Na^+^/K^+^-ATPase with enhancement of SERCA2a-mediated calcium reuptake. Following metabolism, it generates PST3093, a longer-lived metabolite that retains SERCA2a-stimulating properties without significant inhibition of Na^+^/K^+^-ATPase. The structural relationship between istaroxime and PST3093 underlies their distinct pharmacodynamic profiles and contributes to the unique calcium-cycling effects of istaroxime therapy. Abbreviations: Ca^2+^, calcium ion; Na^+^, sodium ion; Na^+^/K^+^-ATPase, sodium–potassium adenosine triphosphatase; NCX, sodium–calcium exchanger; SERCA2a, sarcoplasmic reticulum Ca^2+^-ATPase isoform 2a; SR, sarcoplasmic reticulum; PST3093, istaroxime metabolite with selective SERCA2a-activating properties. ↑ increase. Created in FigureLabs; https://chat.figurelabs.ai/verify/FL-PUB-20260616-SW46HO (accessed on 16 June 2026).

Istaroxime [26,27], (E,Z)-3-[(2-aminoethoxy)imino]androstane-6,17-dione hydrochloride, a derivative of androstenedione, chemically unrelated to cardiac glycosides, has emerged from this pathophysiological reappraisal as a first-in-class agent designed to modulate, rather than simply amplify, intracellular calcium dynamics. By combining inhibition of Na^+^/K^+^-ATPase with enhancement of sarcoplasmic reticulum Ca^2+^-ATPase (SERCA2a) activity, it simultaneously increases calcium availability during systole and facilitates its reuptake during diastole, thereby promoting a more coordinated pattern of calcium cycling [28,29]. The chemical structures of istaroxime and the major metabolite PST3093, followed by the mechanism of action, pharmacodynamic profile, and schematic of the calcium cycling effect, are presented in Figure 1.

Early-phase clinical studies have demonstrated a distinct hemodynamic profile characterized by increased cardiac index, improved diastolic function, and—critically—preservation of heart rate alongside stabilization or elevation of systolic blood pressure [30,31].

In the Hemodynamic Effects of Istaroxime in Patients with Heart Failure (HORIZON-HF) trial [32], istaroxime improved pulmonary capillary wedge pressure, cardiac index, and indices of ventricular relaxation without inducing tachycardia, providing proof of concept for its dual inotropic and lusitropic effects. Subsequent Studies of Istaroxime in Cardiogenic Shock (SEISMiC) [33] extended these findings to patients with hypotensive AHF and early cardiogenic shock, demonstrating increases in systolic blood pressure, stroke volume, and cardiac output without a significant arrhythmic signal. Notably, the ability to enhance contractility while supporting arterial pressure represents a hemodynamic profile that contrasts sharply with that of conventional inotropes, which frequently require concomitant vasopressor therapy.

Importantly, current evidence derives predominantly from patients with hypotensive AHF and pre-cardiogenic shock physiology (corresponding largely to SCAI stage B), and extrapolation of these findings to patients with established cardiogenic shock (SCAI stages C–E), systemic inflammation, severe end-organ hypoperfusion, or multiorgan failure remains speculative.

Despite these promising signals, the current evidence base remains limited to phase II studies focused on hemodynamic endpoints, and the impact of istaroxime on survival, organ function, and progression to advanced shock has not yet been established [34]. Furthermore, no randomized trial has directly compared istaroxime with dobutamine, milrinone, or levosimendan using clinically meaningful outcome measures, limiting assessment of its relative clinical efficacy. As such, its clinical relevance must be interpreted within the broader historical context of inotropic therapy, in which physiological improvement has repeatedly failed to translate into meaningful clinical benefit.

These considerations highlight a critical unmet need for inotropic strategies that are not only haemodynamically effective but also mechanistically aligned with the underlying biology of the failing myocardium. Istaroxime represents a mechanistically distinct approach that aligns with the pathophysiological concept of impaired calcium cycling. Importantly, unlike conventional inotropes, enhancement of SERCA2a function occurs without generalized adrenergic stimulation, suggesting a mechanistic separation between restoring calcium cycling and amplifying calcium overload. Whether this distinction translates into improved clinical outcomes remains unproven and constitutes a key unresolved question.

## 2. Contemporary Pharmacotherapy of Acute Heart Failure

The management of AHF is primarily based on relieving congestion, reducing filling pressures, stabilizing perfusion, and treating precipitating causes. Intravenous loop diuretics remain the cornerstone of therapy, while vasodilators are recommended in patients with preserved blood pressure to improve preload and afterload conditions.

According to the 2023 update of the European Society of Cardiology guidelines [35], inotropes should be reserved for patients with evidence of hypoperfusion or cardiogenic shock and used at the lowest effective dose for the shortest possible duration. This cautious recommendation reflects decades of disappointing experience with traditional inotropes, in which improvements in cardiac output have repeatedly failed to translate into a survival benefit.

The pharmacological limitations of currently available inotropes derive directly from their mechanisms of action. Most agents increase intracellular calcium concentration or adrenergic stimulation, thereby improving contractility at the expense of increased metabolic demand and electrical instability.

Istaroxime emerged from the recognition that the principal abnormality in failing myocardium may not simply be insufficient calcium availability, but rather defective calcium cycling.

## 3. Pathophysiology of Calcium Handling in Acute Heart Failure

The failing myocardium is characterized by profound abnormalities in excitation–contraction coupling. Reduced SERCA2a activity impairs ventricular relaxation, increases diastolic wall tension, reduces sarcoplasmic reticulum calcium stores, and ultimately diminishes systolic calcium release [36,37]. This process contributes simultaneously to systolic dysfunction and diastolic impairment.

Concurrently, chronic sympathetic activation induces β-receptor downregulation and desensitization, reducing responsiveness to adrenergic stimulation. Mitochondrial dysfunction further limits ATP generation, rendering calcium cycling increasingly inefficient and energetically expensive.

Thus, the failing heart is not simply “weak”; it is metabolically exhausted and calcium dysregulated.

Traditional inotropes primarily enhance calcium flux without correcting the underlying defect in calcium recirculation [38,39]. Istaroxime differs fundamentally because it directly targets both calcium availability and calcium reuptake [40].

### 3.1. Molecular Regulation of Calcium Cycling in the Failing Myocardium

At the molecular level, calcium cycling in cardiomyocytes is governed by a tightly regulated network of ion transporters, regulatory proteins, and post-translational modifications that coordinate excitation–contraction coupling. Central to this system is SERCA2a (Sarcoplasmic reticulum Ca^2+^-ATPase isoform 2a), whose activity is dynamically modulated by phospholamban (PLN)—phosphorylated serine 16, which acts as a brake on the calcium cardiac pump (SERCA2a) [41]. In its dephosphorylated state, PLN exerts inhibitory control over SERCA2a, reducing calcium reuptake into the sarcoplasmic reticulum. Phosphorylation of PLN at Ser16 (via protein kinase A) and threonine 17 (Thr17) (via Ca^2+^/calmodulin-dependent protein kinase II, CaMKII) relieves this inhibition, thereby accelerating calcium sequestration and facilitating diastolic relaxation [42,43,44].

In heart failure, this regulatory balance is profoundly disrupted. Reduced SERCA2a expression, increased PLN inhibition, and altered kinase–phosphatase signaling converge to impair calcium reuptake. Concurrently, hyperactivation of CaMKII contributes to pathological phosphorylation of ryanodine receptors (RyR2), increasing diastolic calcium leak from the sarcoplasmic reticulum and promoting arrhythmogenic delayed afterdepolarizations [45,46]. These alterations result in a state characterized not only by reduced systolic calcium availability but also by inefficient calcium recirculation and elevated cytosolic calcium during diastole.

Additional modulation occurs at the level of the sodium–calcium exchanger (NCX), which operates bidirectionally depending on transmembrane sodium and calcium gradients. In failing myocardium, increased intracellular sodium—partly due to reduced Na^+^/K^+^-ATPase activity and late sodium current augmentation—shifts NCX toward reverse-mode operation, contributing to calcium influx during systole but impairing calcium extrusion during diastole [47]. This further exacerbates cytosolic calcium accumulation and disrupts excitation–contraction coupling.

Thus, the molecular phenotype of heart failure is defined not simply by reduced calcium availability, but by dysregulated calcium cycling arising from coordinated disturbances in SERCA2a–PLN interaction, RyR2 stability, CaMKII signaling, and sodium–calcium exchange dynamics [48,49].

### 3.2. Right Ventricular Failure and Pulmonary Circulation Considerations

The hemodynamic profile of acute heart failure is frequently influenced by right ventricular (RV) dysfunction and pulmonary vascular resistance, yet these factors remain underrepresented in discussions of inotropic therapy [50,51]. This omission is clinically relevant, as the optimal pharmacological strategy differs substantially between left- and right-sided failure.

β-adrenergic agonists may improve RV contractility but often increase heart rate, thereby shortening diastolic filling time and impairing coronary perfusion of the right ventricle [52,53]. Phosphodiesterase-3 inhibitors, by contrast, reduce pulmonary vascular resistance and are frequently preferred in RV failure; however, their vasodilatory effects may precipitate systemic hypotension [54]. Levosimendan also exerts pulmonary vasodilatory effects but shares similar hemodynamic limitations [55,56].

The effects of istaroxime on the right ventricle remain incompletely characterized [57]. In the multicenter, randomized, placebo-controlled SEISMiC extension trial [57], in 30 patients with acute decompensated HF-related pre-cardiogenic shock, istaroxime infusion compared with placebo was associated with a greater increase in pulmonary artery compliance and reduction in elastance, suggesting a reduction in right ventricular afterload. Left ventricular contractility improved, and right ventricular contractility stabilized in patients receiving istaroxime, while right ventricular contractility tended to deteriorate over time in the placebo group.

The absence of significant vasodilation and preservation of systemic blood pressure may represent an advantage in maintaining coronary perfusion pressure, particularly in RV ischemia. However, the lack of a strong pulmonary vasodilatory effect suggests that its role in isolated RV failure may be limited compared with PDE-3 inhibitors.

Future studies should specifically address RV–pulmonary coupling, as this may represent a critical determinant of therapeutic differentiation among inotropes.

### 3.3. Myocardial Energetics—Pathophysiology

A central, yet often underemphasized, determinant of inotropic efficacy is myocardial energetics. Calcium cycling is an ATP-dependent process, with SERCA2a-mediated calcium reuptake representing a major component of myocardial energy expenditure. In the failing heart, mitochondrial dysfunction, impaired oxidative phosphorylation, and reduced ATP availability render calcium handling both inefficient and metabolically costly [36,58].

Conventional inotropes exacerbate this imbalance by increasing calcium influx and cycling frequency, thereby amplifying ATP consumption in a system already operating near energetic limits [59,60]. Istaroxime, by enhancing SERCA2a activity, may improve the efficiency of calcium recirculation; however, it does not eliminate the fundamental energy requirement of calcium transport [28,61]. Rather, its potential advantage lies in restoring coordination between calcium release and reuptake, thereby reducing wasted cytosolic calcium accumulation and improving the coupling between energy utilization and contractile work. Whether this translates into a net energetic benefit in vivo remains uncertain and represents a critical area for future investigation. This distinction is particularly relevant when comparing istaroxime with conventional catecholaminergic inotropes. Although SERCA2a activation itself requires ATP, the restoration of coordinated calcium cycling may reduce energetically inefficient calcium accumulation and recirculation within the cytosol. Consequently, the energetic cost associated with enhanced contractility may be lower than that observed with agents that increase calcium influx through cAMP-dependent mechanisms. Nevertheless, current evidence does not conclusively demonstrate improved myocardial energetic efficiency or reduced oxygen consumption, and this hypothesis requires confirmation in dedicated mechanistic studies.

#### Mitochondrial Calcium Handling and Energetic Coupling

Calcium cycling is intimately linked to mitochondrial function, as calcium uptake into mitochondria via the mitochondrial calcium uniporter (MCU) regulates key dehydrogenases in the tricarboxylic acid cycle, thereby modulating ATP production [62,63]. In physiological conditions, transient increases in cytosolic calcium during systole are coupled to mitochondrial calcium uptake, enhancing oxidative phosphorylation in proportion to contractile demand.

In heart failure, this coupling is disrupted. Impaired mitochondrial calcium uptake increased reactive oxygen species (ROS) production, and mitochondrial sodium–calcium exchange (NCLX) dysfunction contributed to reduced ATP generation and energetic inefficiency [64]. At the same time, excessive cytosolic calcium and RyR2-mediated leak increase ATP consumption through futile cycling, further exacerbating the energetic imbalance [65].

**Figure 2:**
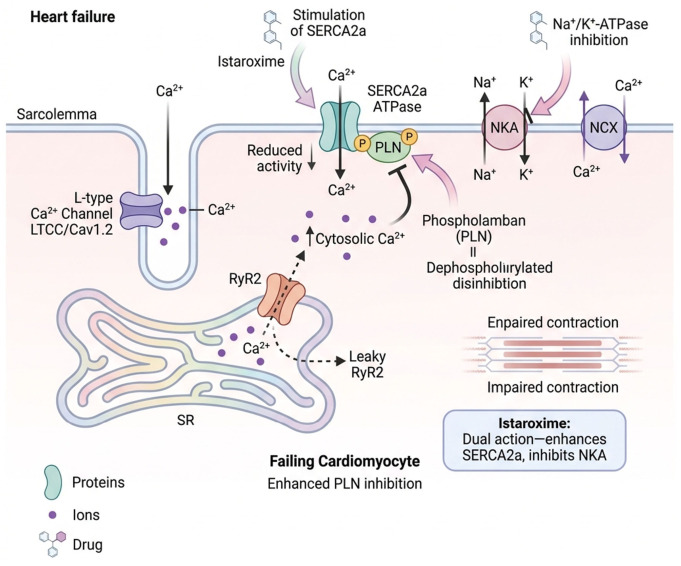
Molecular regulation of calcium cycling in the failing cardiomyocyte and sites of action of istaroxime. In heart failure, impaired calcium handling is characterized by reduced SERCA2a activity, enhanced phospholamban (PLN)-mediated inhibition, and abnormal calcium leakage through ryanodine receptors (RyR2), resulting in elevated cytosolic calcium, impaired sarcoplasmic reticulum (SR) calcium storage, and reduced contractile performance. Istaroxime exerts a dual mechanism of action by stimulating SERCA2a activity, thereby enhancing calcium reuptake into the SR during diastole, and by inhibiting the Na^+^/K^+^-ATPase (NKA), which increases intracellular sodium and, in turn, augments calcium availability by modulating the sodium–calcium exchanger (NCX). Together, these effects improve calcium cycling, restore SR calcium stores, enhance systolic contraction, and support diastolic relaxation in the failing myocardium. Abbreviations: LTCC/Cav1.2, L-type calcium channel; NCX, sodium–calcium exchanger; NKA, Na^+^/K^+^-ATPase; PLN, phospholamban; RyR2, ryanodine receptor type 2; SERCA2a, sarcoplasmic reticulum Ca^2+^-ATPase isoform 2a; SR, sarcoplasmic reticulum. ↑ increase; ↓ decrease. Created in FigureLabs; https://chat.figurelabs.ai/verify/FL-PUB-20260615-GTGRSW (accessed on 16 June 2026).

By improving the coordination of calcium release and reuptake, istaroxime may indirectly influence mitochondrial calcium dynamics and energetic efficiency. However, whether enhanced SERCA2a activity translates into improved mitochondrial coupling or merely redistributes energetic demand remains uncertain and represents an important area for future investigation. Molecular regulation of calcium cycling in the falling cardiomyocyte and sites of isatroxime action presents Figure 2.

## 4. Mechanisms of Action of Contemporary Inotropes: Molecular Pathways, Hemodynamic Expression, and Clinical Translation

The pharmacological augmentation of myocardial contractility in acute heart failure has historically relied on modulation of intracellular calcium dynamics [66,67]. However, the manner in which calcium is mobilized, redistributed, and energetically supported differs substantially across drug classes. These mechanistic differences are not merely biochemical—they determine hemodynamic profiles, arrhythmogenic risk, and, ultimately, the success or failure of clinical translation. A detailed understanding of each class, therefore, requires integrating molecular signaling, cellular physiology, and trial-derived clinical observations.

**Figure 3:**
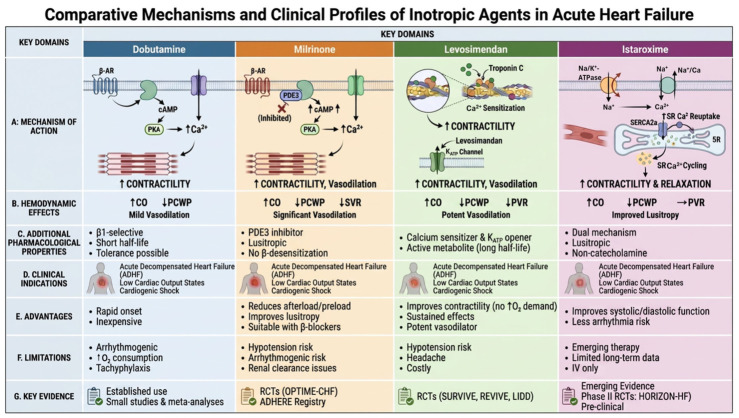
Comparative profile of inotropic agents in acute heart failure. A structured comparison of contemporary inotropic agents, including istaroxime, levosimendan, dobutamine, and milrinone, is presented across key domains: mechanism of action, hemodynamic effects, additional pharmacological properties, clinical indications, advantages, limitations, and available evidence. Conventional inotropes (dobutamine and milrinone) primarily enhance contractility through cyclic AMP–mediated calcium amplification, resulting in robust increases in cardiac output but at the cost of increased myocardial oxygen demand and arrhythmogenic potential. Levosimendan improves contractile efficiency through calcium sensitization and vasodilation, while istaroxime uniquely combines increased calcium availability with enhanced calcium reuptake, promoting coordinated calcium cycling. These mechanistic differences translate into distinct hemodynamic profiles, safety considerations, and potential phenotype-specific applications. Abbreviations: AC, adenylyl cyclase; ADHF, acute decompensated heart failure; β-AR, β-adrenergic receptor; cAMP, cyclic adenosine monophosphate; CO, cardiac output; K_ATP_, ATP-sensitive potassium channel; Na^+^/Ca^2+^, sodium–calcium exchanger; Na^+^/K^+^-ATPase, sodium–potassium adenosine triphosphatase; O_2_, oxygen; PCWP, pulmonary capillary wedge pressure; PDE3, phosphodiesterase type 3; PKA, protein kinase A; PVR, pulmonary vascular resistance; RCT, randomized controlled trial; SERCA2a, sarcoplasmic reticulum Ca^2+^-ATPase isoform 2a; SR, sarcoplasmic reticulum; SVR, systemic vascular resistance; TnC, troponin C. Clinical trials: ADHERE, Acute Decompensated Heart Failure National Registry; HORIZON-HF, Hemodynamic, Echocardiographic, and Neurohormonal Effects of Istaroxime, a Novel Intravenous Inotropic and Lusitropic Agent; LIDO, Levosimendan Infusion versus Dobutamine Study; OPTIME-CHF, Outcomes of a Prospective Trial of Intravenous Milrinone for Exacerbations of Chronic Heart Failure; REVIVE, Randomized Evaluation of Intravenous Levosimendan Efficacy; SURVIVE, Survival of Patients with Acute Heart Failure in Need of Intravenous Inotropic Support. ↑ increase; ↓ decrease. Created in FigureLabs; https://chat.figurelabs.ai/verify/FL-PUB-20260623-JQ5F43 (accessed on 16 June 2026).

The heterogeneity of inotropic agents is best understood through direct comparison of their underlying mechanisms, hemodynamic effects, and clinical profiles (Figure 3). While conventional agents such as dobutamine and milrinone increase contractility by amplifying intracellular calcium flux, this approach is associated with increased myocardial oxygen consumption and arrhythmogenic risk. Levosimendan improves contractile efficiency without increasing calcium concentration but is limited by vasodilatory effects and prolonged pharmacodynamic effects. In contrast, istaroxime integrates enhancement of calcium availability with improved sarcoplasmic reticulum reuptake, resulting in a more coordinated pattern of calcium cycling. These differences highlight that inotropic therapies are not interchangeable, and their selection should be guided by both mechanistic properties and hemodynamic context.

### 4.1. β-Adrenergic Agonists: cAMP-Driven Amplification of Calcium Cycling

β-adrenergic agonists, exemplified by dobutamine, exert their effects by stimulating β1-adrenergic receptors coupled to Gs proteins, leading to activation of adenylate cyclase and increased intracellular cyclic AMP (cAMP) [68]. Protein kinase A phosphorylates multiple components of the excitation–contraction coupling machinery, including L-type calcium channels, ryanodine receptors, phospholamban, and troponin I. The net effect is a marked increase in calcium turnover, resulting in enhanced inotropy and lusitropy, accompanied by increased heart rate and conduction velocity.

From a physiological standpoint, this represents a high-flux, high-energy state. The myocardium is forced to cycle calcium more rapidly and at greater amplitude, which significantly increases ATP consumption. Importantly, this occurs in a metabolic environment already characterized by mitochondrial dysfunction and impaired oxidative phosphorylation.

This mechanism explains several consistent clinical observations, including increased myocardial oxygen demand related to tachycardia, arrhythmogenesis, driven by diastolic calcium leak and delayed afterdepolarizations, and diminished efficacy in chronic heart failure, due to β-receptor downregulation.

Clinical data support these mechanistic liabilities. Observational registries and randomized comparisons have consistently failed to demonstrate survival benefit, while reporting increased arrhythmic events. In the DOREMI trial [21], dobutamine did not differ from milrinone in mortality or major outcomes, reinforcing the concept that cAMP-mediated calcium amplification—regardless of the upstream trigger—does not translate into improved prognosis.

Thus, β-agonists can be understood as potent but metabolically inefficient amplifiers of contractility, whose clinical utility is confined to short-term hemodynamic rescue. These limitations provide the rationale for alternative approaches such as istaroxime, which seeks to improve contractility without relying on sustained adrenergic stimulation.

### 4.2. Phosphodiesterase-3 Inhibitors: Receptor-Independent cAMP Accumulation

Phosphodiesterase-3 (PDE-3) inhibitors, such as milrinone, achieve similar downstream effects via distinct upstream mechanisms. By inhibiting PDE-3, they prevent the degradation of cAMP, leading to sustained activation of PKA independently of β-receptor signaling [69,70].

This distinction has practical implications, including preserved efficacy in patients receiving β-blockers, reduced reliance on adrenergic tone, and more pronounced effects on vascular smooth muscle.

At the cellular level, however, the consequences remain largely the same as with β-agonists: increased calcium influx, enhanced sarcoplasmic reticulum release, and accelerated cycling. Thus, PDE-3 inhibition represents not a fundamentally different mechanism, but rather an alternative route to sustained cAMP elevation.

A key differentiating feature is the vasodilatory effect, mediated by cAMP in vascular smooth muscle. This leads to reductions in systemic and pulmonary vascular resistance, which may be beneficial in right ventricular failure, pulmonary hypertension, and high afterload states.

However, this vasodilation introduces a critical limitation: hypotension, particularly in patients with marginal perfusion.

The clinical equivalence of PDE-3 inhibitors and β-agonists was clearly demonstrated in the DOREMI trial [21], which showed no difference in mortality or composite outcomes between milrinone and dobutamine in cardiogenic shock. This finding is mechanistically coherent: despite different receptor-level pathways, both drugs converge on cAMP-driven calcium overload and increased metabolic demand, thereby sharing the same fundamental limitations.

Thus, PDE-3 inhibitors may be best understood as β-independent amplifiers of the same energetically costly pathway, with additional vascular effects that may be either beneficial or deleterious depending on hemodynamic context. Unlike PDE-3 inhibitors, istaroxime does not cause significant systemic vasodilation and may therefore help preserve arterial pressure in hypotensive patients.

### 4.3. Calcium Sensitizers: Modulation of Contractile Efficiency

Calcium sensitizers, exemplified by levosimendan, represent an alternative approach to inotropic therapy that does not rely on increasing intracellular calcium concentration. Instead, levosimendan enhances the sensitivity of troponin C to calcium, thereby facilitating actin–myosin interaction at existing calcium levels and improving contractile efficiency [71,72].

In parallel, levosimendan activates ATP-sensitive potassium (K-ATP) channels in vascular smooth muscle and mitochondria, leading to systemic and pulmonary vasodilation and potentially exerting cardioprotective effects by stabilizing mitochondria [73,74]. This dual mechanism seeks to partially decouple contractility from calcium overload, theoretically reducing the energetic cost and arrhythmogenic risk associated with calcium amplification.

However, the clinical translation of this mechanism reveals important limitations. The vasodilatory component is often dominant, predisposing to hypotension, particularly in hemodynamically unstable patients. In addition, the presence of active metabolites results in prolonged hemodynamic effects, limiting titratability in acute settings. Finally, because its efficacy depends on existing intracellular calcium availability, levosimendan may be less effective in advanced states characterized by impaired calcium handling and depleted sarcoplasmic reticulum stores.

Clinical trials reflect these mechanistic constraints. While studies such as Randomized Evaluation of Intravenous Levosimendan Efficacy II (REVIVE II) [23] demonstrated improvements in symptoms and haemodynamics, no consistent survival benefit has been observed, and trials such as Survival of Patients with Acute Heart Failure in Need of Intravenous Inotropic Support (SURVIVE) trial [22] failed to demonstrate superiority over conventional inotropes. These findings suggest that improving contractile efficiency alone, without correcting underlying abnormalities in calcium cycling and energetics, may be insufficient to alter clinical outcomes. In contrast to levosimendan, istaroxime directly targets abnormalities in calcium recirculation by modulating SERCA2a.

### 4.4. Istaroxime: Dual Modulation of Calcium Availability and Reuptake

Istaroxime introduces a mechanistically distinct approach by simultaneously influencing calcium availability and calcium recirculation, targeting a central abnormality in the failing myocardium.

Its pharmacological action consists of two coordinated components. First, inhibition of Na^+^/K^+^-ATPase increases intracellular sodium concentration, thereby reducing sodium–calcium exchanger (NCX) activity and augmenting intracellular calcium availability during systole [75]. This effect resembles digitalis-like inotropy but is characterized by a shorter duration of action and greater titratability.

Second, and more distinctively, istaroxime enhances SERCA2a function through modulation of phospholamban, promoting more rapid reuptake of calcium into the sarcoplasmic reticulum during diastole. This leads to reduced cytosolic calcium accumulation, increased sarcoplasmic reticulum calcium stores, and improved coordination between diastolic relaxation and subsequent systolic contraction.

Although β-adrenergic agonists and phosphodiesterase-3 inhibitors also accelerate SERCA2a activity through protein kinase A–mediated phosphorylation of phospholamban, this effect occurs in the context of global cAMP activation and substantial amplification of calcium influx. Consequently, improved calcium reuptake is accompanied by increased intracellular calcium loading, greater myocardial oxygen demand, and enhanced arrhythmogenic potential. In contrast, istaroxime promotes SERCA2a-mediated calcium recirculation without relying on adrenergic stimulation or sustained cAMP elevation. This distinction may allow more efficient restoration of excitation–contraction coupling while limiting the excessive calcium flux that characterizes conventional inotropic therapy.

The net effect is not simply amplification of calcium flux, but partial restoration of physiological calcium cycling. This distinction has several important implications. Enhanced lusitropy addresses a key limitation of conventional inotropes, while reducing diastolic calcium accumulation may mitigate arrhythmogenic potential. In addition, the absence of adrenergic stimulation allows preservation of heart rate, and the minimal vasodilatory effect may contribute to stabilization or modest increases in arterial pressure.

### 4.5. Molecular Basis of Istaroxime Action on SERCA2a and Na^+^/K^+^-ATPase

The dual mechanism of istaroxime reflects its interaction with two critical molecular systems governing intracellular ion homeostasis [76]. Its inhibition of Na^+^/K^+^-ATPase is mediated by binding to the enzyme’s α-subunit, leading to increased intracellular sodium concentration. This alters the electrochemical gradient that drives NCX activity, favoring reduced calcium extrusion and thereby augmenting cytosolic calcium availability during systole.

In parallel, istaroxime enhances SERCA2a function by modulating phospholamban-mediated inhibition [61]. Although the precise molecular interaction remains incompletely characterized, experimental data suggest that istaroxime promotes a conformational state of SERCA2a that reduces PLN inhibitory binding, functionally mimicking the effects of PLN phosphorylation [61]. Unlike cAMP-mediated inotropes, this effect is achieved without generalized activation of downstream adrenergic signaling pathways, which may contribute to a more favorable balance between inotropy, lusitropy, and arrhythmic risk. This results in accelerated calcium reuptake into the sarcoplasmic reticulum, increased calcium store loading, and improved coordination between diastolic relaxation and subsequent systolic release.

Importantly, this dual modulation distinguishes istaroxime from both cardiac glycosides and cAMP-mediated inotropes. Unlike digoxin, which increases intracellular calcium without enhancing reuptake, istaroxime restores bidirectional calcium flux. Unlike β-adrenergic stimulation, it does not rely on upstream receptor signaling or global kinase activation, potentially avoiding maladaptive phosphorylation cascades associated with chronic adrenergic drive.

Early clinical studies are consistent with this mechanistic profile. Improvements in cardiac index, filling pressures, and diastolic parameters have been observed without significant tachycardia, while increases in systolic blood pressure have been reported in hypotensive populations [34]. However, these findings remain limited to phase II studies, and their translation into improved clinical outcomes has not yet been established.

### 4.6. Istaroxime in Relation to Cardiac Glycosides

The inhibition of Na^+^/K^+^-ATPase by istaroxime invites comparison with cardiac glycosides, particularly digoxin. Both agents increase intracellular sodium concentration, thereby enhancing calcium availability by reducing the activity of the sodium–calcium exchanger [77]. However, their downstream effects differ substantially.

Digoxin does not directly influence SERCA2a function and therefore does not facilitate calcium reuptake into the sarcoplasmic reticulum. As a result, it increases intracellular calcium without restoring coordinated calcium cycling, contributing to its narrow therapeutic index and well-established arrhythmogenic potential.

In contrast, istaroxime combines Na^+^/K^+^-ATPase inhibition with enhancement of calcium reuptake, promoting both systolic calcium release and diastolic sequestration. This integrated mechanism may provide a more balanced modulation of excitation–contraction coupling.

Pharmacokinetic differences further distinguish the two agents. Digoxin is characterized by a long half-life and tissue accumulation, limiting its utility in acute haemodynamic management. Istaroxime, by contrast, is short-acting, with a half-life of less than 1 h, and administered as a continuous infusion, allowing rapid titration and withdrawal of effect [76]. A terminal istaroxime metabolite has a longer half-life, approximately 14 h, and acts as a selective SERCA2a activator.

Thus, although both agents share a common molecular target, istaroxime is more appropriately viewed as a mechanistic evolution rather than a direct analogue of cardiac glycosides. Additional differences extend beyond molecular pharmacology. Digoxin possesses a narrow therapeutic window, is highly dependent on renal clearance, and is associated with clinically significant toxicity in the setting of renal dysfunction, electrolyte abnormalities, or drug interactions. In contrast, istaroxime is administered as a short-term intravenous infusion with rapid onset and offset of action, allowing greater titratability in acute hemodynamic management. While both agents share Na^+^/K^+^-ATPase inhibition as a central mechanism, their pharmacokinetic profiles, safety considerations, and intended clinical applications differ substantially.

### 4.7. Calcium Cycling, Calcium Transients, and Electrophysiological Implications

The electrophysiological consequences of inotropic therapy are closely linked to their effects on intracellular calcium dynamics. Conventional inotropes increase calcium amplitude and promote diastolic calcium leak, predisposing to delayed afterdepolarizations and ventricular arrhythmias.

By enhancing SERCA2a-mediated calcium reuptake, istaroxime may reduce diastolic cytosolic calcium accumulation and promote more synchronized calcium transients. This provides a plausible mechanistic basis for a lower arrhythmogenic profile compared with agents that amplify calcium influx [78].

However, the concomitant inhibition of Na^+^/K^+^-ATPase introduces additional complexity. Alterations in intracellular sodium concentration may influence membrane excitability and calcium exchange dynamics, with potential electrophysiological consequences that are not yet fully characterized.

While early clinical studies have not demonstrated a significant proarrhythmic signal, these observations are based on limited sample sizes and controlled conditions. A definitive assessment of electrophysiological safety will require larger studies with broader patient populations and longer follow-up.

### 4.8. Molecular Determinants of Arrhythmogenesis in Calcium Dysregulation

At the molecular level, arrhythmogenesis in heart failure is closely linked to abnormal calcium handling and ion channel remodeling [79,80,81]. Diastolic calcium leak through hyperphosphorylated RyR2 channels leads to spontaneous calcium release events, which activate the sodium–calcium exchanger in forward mode, generating transient inward currents that underlie delayed afterdepolarizations.

In parallel, alterations in intracellular sodium concentration influence membrane excitability by affecting NCX activity and action potential duration [82,83]. Increased late sodium current (I_Na, L) further contributes to intracellular sodium accumulation, amplifying calcium overload and electrical instability [84].

By enhancing SERCA2a-mediated calcium reuptake and reducing cytosolic calcium persistence during diastole, istaroxime may attenuate the substrate for delayed afterdepolarizations [78,85]. However, its simultaneous inhibition of Na^+^/K^+^-ATPase introduces competing effects on intracellular sodium dynamics, with the net electrophysiological consequences remaining incompletely defined.

### 4.9. Comparative Mechanistic Perspective

Current inotropic therapies can be broadly categorized into distinct strategies for modulating myocardial contractility, each defined by its interaction with intracellular calcium handling.

β-adrenergic agonists and phosphodiesterase-3 inhibitors increase contractility by amplifying intracellular calcium flux, a mechanism that is effective but is associated with increased metabolic demand and arrhythmogenic risk. Calcium sensitizers enhance contractile efficiency without increasing calcium concentration but do not address impaired calcium recirculation and are limited by vasodilatory effects.

Istaroxime differs in that it combines augmentation of calcium availability with enhancement of calcium reuptake, promoting a more coordinated pattern of calcium cycling. Importantly, this mechanism should not be interpreted as energetically neutral. SERCA2a-mediated calcium reuptake remains ATP dependent, and the apparent energetic advantage of istaroxime may arise from improved efficiency of calcium handling rather than a reduction in absolute energy expenditure. This integrated mechanism is theoretically aligned more closely with the underlying pathophysiology of heart failure, in which impaired calcium reuptake plays a central role. However, mechanistic plausibility should not be equated with clinical efficacy. Previous inotropic therapies have frequently demonstrated favorable physiological effects without producing improvements in long-term outcomes. Whether restoration of calcium cycling translates into meaningful clinical benefit remains unknown.

Despite this mechanistic distinction, it remains uncertain whether restoration of calcium cycling translates into meaningful clinical benefit. Historical experience with inotropic therapy has consistently demonstrated that improvements in haemodynamic parameters do not necessarily lead to improved outcomes.

Accordingly, the potential value of istaroxime lies not only in its mechanistic novelty, but in its ability—yet to be demonstrated—to influence the clinical trajectory of acute heart failure beyond short-term haemodynamic improvement.

The comparative features of these agents—including their effects on calcium dynamics, heart rate, vascular resistance, arrhythmogenic potential, and energetic efficiency—are summarized in Table 1.

**Figure 4:**
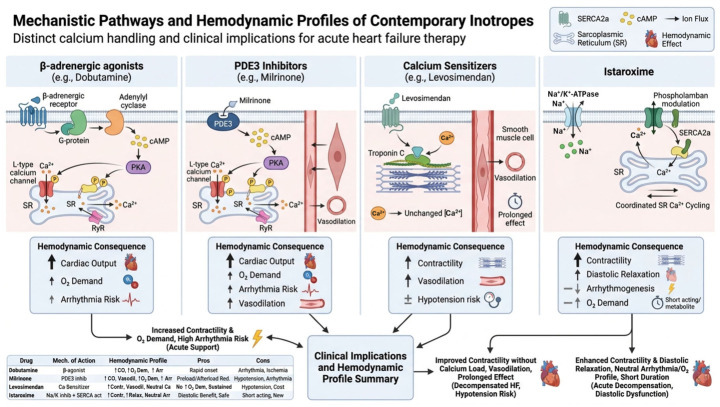
Mechanistic pathways and hemodynamic profiles of contemporary inotropes. Contemporary inotropic agents exert their effects through distinct mechanisms of intracellular calcium modulation. β-adrenergic agonists (e.g., dobutamine) and phosphodiesterase-3 inhibitors (e.g., milrinone) increase contractility by amplifying cyclic AMP–mediated calcium influx and sarcoplasmic reticulum release, thereby increasing calcium flux and the energetic demand, with an increased arrhythmogenic potential. Calcium sensitizers (e.g., levosimendan) enhance contractile efficiency without increasing intracellular calcium concentration but are limited by vasodilatory effects and prolonged pharmacodynamics. In contrast, istaroxime combines Na^+^/K^+^-ATPase inhibition with enhancement of SERCA2a-mediated calcium reuptake, promoting a more coordinated pattern of calcium cycling that integrates systolic augmentation with improved diastolic relaxation. These mechanistic differences translate into distinct hemodynamic profiles and may underlie differential clinical applicability across hemodynamic phenotypes. Abbreviations: β, beta (adrenergic receptor); Ca^2+^, calcium ion; cAMP, cyclic adenosine monophosphate; O_2_, oxygen; PDE3, phosphodiesterase type 3; PKA, protein kinase A; RyR, ryanodine receptor; SERCA2a, sarcoplasmic reticulum Ca^2+^-ATPase isoform 2a; SR, sarcoplasmic reticulum. Arr, arrhythmia risk; Cont, contractility; Dem, myocardial oxygen demand; HR, heart rate; Vasodil, vasodilation. ↑ increase; ± variable effects; ↓ decrease; - no change. Created in FigureLabs; https://chat.figurelabs.ai/verify/FL-PUB-20260616-WYJGBN (accessed on 16 June 2026).

The mechanistic differences among contemporary inotropic agents are summarized in Figure 4. While β-adrenergic agonists and phosphodiesterase-3 inhibitors converge on amplification of intracellular calcium flux, and calcium sensitizers primarily enhance contractile efficiency, istaroxime introduces a distinct paradigm by modulating both calcium availability and reuptake. This integrated mechanism results in a haemodynamic profile that differs qualitatively from conventional agents and may be more closely aligned with the underlying pathophysiology of impaired calcium cycling in heart failure.

## 5. Clinical Evidence Supporting Istaroxime

The randomized, multicenter, double-blind, placebo-controlled, dose-escalation, phase II HORIZON-HF trial (Hemodynamic, Echocardiographic, and Neurohormonal Effects of Istaroxime, a Novel Intravenous Inotropic and Lusitropic Agent: a Randomized Controlled Trial in Patients Hospitalized with Heart Failure) [32] included 120 patients randomized 3:1 to istaroxime (doses of 0.5 µg/kg/min, 1.0 µg/kg/min, or 1.5 µg/kg/min) or placebo infusion, followed by pulmonary artery catheterization and echocardiography. The primary endpoint was the change in pulmonary capillary wedge pressure (PCWP) compared to placebo after a 6 h continuous infusion. The study first demonstrated that istaroxime improved pulmonary capillary wedge pressure, cardiac index, and diastolic function without increasing heart rate. Compared with placebo, istaroxime improved almost all measured hemodynamic parameters. The most important finding was a statistically significant decrease in PCWP, accompanied by an increase in systolic blood pressure. On echocardiography, although conventional parameters of systolic performance did not change substantially, regional myocardial tissue velocity (S′) improved significantly, suggesting that istaroxime enhances both regional and global cardiac contractility. Diastolic stiffness decreased with increased istaroxime doses, as reflected by increased E′ velocities and decreased E/A and E/E′ ratios. The increase in E′ occurred in parallel with an increase in systolic blood pressure, which is a remarkable and uncommon combination among inotropic therapies. Subsequent analyses suggested improvements in ventricular stiffness and relaxation dynamics.

In the Istaroxime ADHF Trial, a randomized, multicenter, double-blind, placebo-controlled phase II trial [86], a 24 h infusion of istaroxime at 0.5 or 1.0 µg/kg/min was compared to placebo, in 120 patients hospitalized for acute HF with reduced left ventricular ejection fraction. The primary efficacy endpoint was the change in diastolic parameters on echocardiography from baseline to 24 h after the start of the infusion, reflected by the E/e’ ratio. The study showed that istaroxime at 0.5 or 1.0 µg/kg/min improves left ventricular diastolic and systolic function without increasing major cardiac adverse events. The primary endpoint, E/e’ratio, was significantly reduced with both doses of istaroxime, and moreover, stroke volume index increased. An improvement was observed in other echocardiographic parameters, including the E/A ratio, left atrial dimensions, and inferior vena cava diameter. Changes in the left ventricular ejection fraction and left ventricular volumes did not reach statistical significance. Renal function, measured by estimated glomerular filtration rate, increased with both istaroxime doses, whereas in the placebo group a decrease was observed. A 24 h Holter monitoring did not reveal differences in clinically significant arrhythmias.

More recent SEISMiC studies [34,57] evaluated istaroxime in patients with AHF-related pre-cardiogenic shock characterized by hypotension without overt hypoperfusion. Istaroxime significantly improved systolic blood pressure, cardiac index, stroke volume, and echocardiographic parameters while maintaining a favourable safety profile.

In the randomized, multicenter, double-blind, placebo-controlled, Phase II SEISMiC trial (The Safety and Efficacy of Istaroxime for Pre-Cardiogenic Shock) (Part A) [34], in a cohort of 60 patients, istaroxime (at the dose of 1.0–1.5 µg/kg/min) compared to placebo, showed a beneficial effect on blood pressure, and at 24 h, improvement in echocardiographic measurements, including cardiac index, left atrial area, and left ventricular end-systolic volume. There were no significant differences in pulse pressure, serious adverse events, or adverse events, except for more nausea, vomiting, and infusion site pain in istaroxime-treated patients.

In the SEISMiC—extension study [87], the effects of intravenous istaroxime infusion at 0.5 to 1.5 µg/kg/min for up to 60 h were compared to placebo in 90 patients with SCAI stage B cardiogenic shock related to acute HF. Istaroxime was associated with a greater increase in systolic blood pressure, which persisted for 60 h when infusion was continued for at least 48 h. Treatment with istaroxime also increased cardiac output (by 0.66 L/min, *p* = 0.017), and reduced PCWP (by 3.8 mmHg, *p* = 0.0017). On echocardiography, improvements were observed in the E/A ratio, tricuspid annular plane systolic excursion (TAPSE), and left atrial volume at 24 h. Heart rate decreased, and no significant malignant arrhythmias were detected on Holter monitoring in the istaroxime group.

In the post hoc analysis of the SEISMiC extension trial [57], invasive hemodynamic indices and echocardiographic parameters were subsequently compared in 30 patients with SCAI stage B cardiogenic shock related to acute decompensated HF. Istaroxime infusion led to sustained improvement in aortic pulsatility index and left ventricular stroke work index, along with increased pulmonary arterial compliance and reduced pulmonary arterial elastance at 48 h. Pressure–volume loop analysis showed that, in the placebo group, left ventricular contractility remained largely stable while right ventricular contractility tended to deteriorate over time, whereas in the istaroxime group, left ventricular contractility improved and right ventricular contractility was essentially preserved. The increase in both left ventricular and right ventricular end-systolic elastance from baseline was greater with istaroxime than with placebo, indicating concurrent augmentation of left ventricular systolic function and protection of right ventricular function in the setting of early cardiogenic shock.

A particularly important observation across studies is the absence of substantial tachycardia or excess arrhythmias, contrasting sharply with traditional catecholaminergic inotropes.

Furthermore, istaroxime appears capable of increasing blood pressure while improving contractility—a highly unusual hemodynamic profile in acute heart failure pharmacology. Despite these encouraging hemodynamic observations, no study to date has demonstrated improvements in mortality, rehospitalization, progression to advanced cardiogenic shock, duration of intensive care, or other hard clinical outcomes. Consequently, the available evidence should be interpreted as demonstrating physiological efficacy rather than proven clinical superiority.

While mechanistic considerations provide a necessary framework for understanding inotropic therapy, their clinical relevance ultimately depends on translation into haemodynamic improvement, safety, and patient-centered outcomes. The clinical development of istaroxime has therefore focused on evaluating whether its dual modulation of calcium influx and reuptake results in a haemodynamic profile that differs meaningfully from conventional agents. Early-phase trials have consistently assessed not only traditional parameters such as cardiac output and filling pressures, but also indices of diastolic function, heart rate response, and blood pressure stability—domains in which istaroxime is mechanistically expected to demonstrate advantage. Importantly, these studies have been conducted in carefully selected populations, often representing early or pre-shock states, where modulation of calcium cycling may still be biologically effective. The principal clinical investigations of istaroxime, together with their key hemodynamic findings and mechanistic implications, are summarized in Table 2.

While these studies provide consistent haemodynamic signals, their clinical interpretation requires integration with mechanistic context and recognition of important limitations, which are discussed below.

## 6. Translational Interpretation and Phenotype-Based Application of Istaroxime

The clinical development of istaroxime has been characterized by a consistent focus on haemodynamic endpoints, reflecting its mechanistic premise of restoring calcium cycling rather than simply augmenting contractility. Across early-phase studies, a coherent pattern has emerged: improvement in cardiac performance accompanied by preservation of heart rate and stabilization of arterial pressure.

These findings distinguish istaroxime from conventional inotropes not by the magnitude of cardiac output augmentation, but by the qualitative nature of the haemodynamic response. β-adrenergic agonists and phosphodiesterase-3 inhibitors increase cardiac output primarily through amplification of intracellular calcium flux, often at the expense of tachycardia, increased myocardial oxygen demand, and haemodynamic instability. Calcium sensitizers improve contractile efficiency but frequently introduce vasodilatory effects that may be poorly tolerated in hypotensive states. In contrast, istaroxime appears to enhance stroke volume while maintaining heart rate and supporting or increasing systolic blood pressure—a profile that does not conform to traditional pharmacological trade-offs.

**Figure 5:**
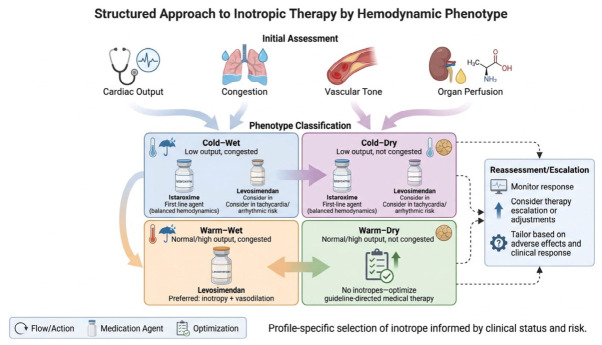
Conceptual framework for phenotype-guided inotropic therapy in acute heart failure. The figure illustrates a hypothetical approach to selecting inotropic support according to hemodynamic phenotype. The proposed framework is based on currently available mechanistic and clinical data and should be regarded as conceptual rather than evidence based. No randomized trial has established the superiority of istaroxime over conventional inotropes in any specific hemodynamic phenotype. A structured conceptual approach to inotropic therapy based on hemodynamic phenotype is illustrated. Initial assessment integrates cardiac output, congestion, vascular tone, and markers of organ perfusion to classify patients into four profiles: cold–wet, cold–dry, warm–wet, and warm–dry. In low-output states (cold–wet and cold–dry), istaroxime is proposed as a first-line agent due to its balanced hemodynamic profile, with levosimendan considered in the presence of tachycardia or arrhythmic risk. In patients with preserved output and predominant congestion (warm–wet), levosimendan may be preferred due to its combined inotropic and vasodilatory effects. In warm–dry patients, inotropic therapy is generally not indicated, and optimization of guideline-directed medical therapy is prioritized. The algorithm also incorporates reassessment, escalation strategies, and expected pharmacodynamic effects of each agent. This figure represents a mechanistic and conceptual framework rather than an evidence-based treatment algorithm and should not be interpreted as demonstrating clinical superiority of one agent over another. Created in FigureLabs; https://chat.figurelabs.ai/verify/FL-PUB-20260616-W01VO8 (accessed on 16 June 2026).

A phenotype-guided approach provides a rational framework for inotropic selection in acute heart failure (Figure 5). Rather than applying inotropes uniformly, therapy is aligned with the underlying hemodynamic profile, integrating cardiac output, congestion, and vascular tone [88,89]. Patients with low-output states (cold–wet and cold–dry) are most likely to benefit from agents that improve contractility without exacerbating tachycardia or hypotension, a profile that may favor istaroxime. In contrast, patients with preserved output and predominant congestion (warm–wet) may derive greater benefit from vasodilatory inotropes such as levosimendan. In stabilized patients without congestion or hypoperfusion (warm–dry), inotropic therapy is generally not indicated. This framework emphasizes that the effectiveness of inotropic therapy is phenotype-dependent and supports a more individualized, physiology-driven treatment strategy.

### 6.1. Haemodynamic Signal and Mechanistic Coherence

Early clinical studies have demonstrated that istaroxime improves cardiac index, reduces filling pressures, and enhances indices of diastolic function without inducing tachycardia. These effects are mechanistically consistent with coordinated modulation of calcium availability and reuptake. Enhancement of SERCA2a-mediated calcium sequestration during diastole may reduce cytosolic calcium accumulation, improve ventricular relaxation, and facilitate more efficient systolic calcium release.

Importantly, studies conducted in hypotensive acute heart failure and early cardiogenic shock [34,57] have reported increases in systolic blood pressure alongside improvements in stroke volume and cardiac output. This combination is uncommon among inotropic agents, which typically require concomitant vasopressor support to maintain arterial pressure. The apparent ability of istaroxime to support both perfusion and pressure suggests a haemodynamic profile that may be particularly relevant in early haemodynamic compromise.

However, these observations must be interpreted within the constraints of early-phase clinical investigation. Available studies are limited in size, duration, and patient heterogeneity, and are not designed to assess clinical outcomes.

### 6.2. Phenotype Dependence and Biological Plausibility

A critical feature of the existing evidence is the selection of specific haemodynamic phenotypes. Clinical trials of istaroxime have predominantly enrolled patients with acute heart failure characterized by hypotension or early cardiogenic shock, often corresponding to Society for Cardiovascular Angiography and Interventions (SCAI) stages A–B [90,91].

This phenotype selection is mechanistically coherent. The therapeutic premise of istaroxime—restoration of calcium cycling through SERCA2a modulation—presupposes the presence of viable myocardium with preserved, albeit impaired, excitation–contraction coupling. In advanced cardiogenic shock, where mitochondrial dysfunction, systemic inflammation, and cellular injury are profound, modulation of intracellular calcium handling alone may be insufficient to restore meaningful contractile function.

Accordingly, current evidence suggests that istaroxime may be most relevant in early or evolving haemodynamic compromise (SCAI A–B), hypotensive acute heart failure where maintenance of arterial pressure is critical, and mixed systolic–diastolic dysfunction, in which impaired relaxation contributes to instability.

In contrast, its role in advanced shock (SCAI C–E) remains uncertain, where pharmacological inotropy is frequently insufficient and mechanical circulatory support becomes central to management.

### 6.3. Clinical Positioning Within the Inotropic Landscape

The selection of inotropic therapy in acute heart failure is inherently based on balancing competing haemodynamic priorities, including cardiac output, arterial pressure, vascular tone, and heart rate. Existing agents achieve these effects through trade-offs. β-adrenergic agonists provide rapid augmentation of cardiac output but at the cost of tachycardia and increased metabolic demand. Phosphodiesterase-3 inhibitors offer β-independent inotropy and pulmonary vasodilation but frequently induce hypotension. Calcium sensitizers improve contractile efficiency but are limited by vasodilatory effects and prolonged pharmacodynamics.

Istaroxime introduces a haemodynamic profile that does not fit within these traditional categories. Its ability to augment stroke volume while preserving heart rate and stabilizing blood pressure suggests a potential role in patients who are poorly served by existing pharmacological options, particularly those in whom hypotension limits the use of vasodilatory agents.

From a phenotype-based perspective, istaroxime may therefore occupy a distinct therapeutic niche rather than replacing established inotropes. In early haemodynamic compromise, it may provide stabilization without the need for high-dose catecholamines or combined vasopressor–inotrope strategies. In more advanced disease, it may serve as a bridging therapy, although this role remains speculative.

### 6.4. Translational Implications and Unresolved Questions

A recurring and unresolved paradox in acute heart failure is the consistent failure of mechanistically sound inotropic strategies to translate into improved clinical outcomes. Across multiple drug classes, interventions grounded in robust physiological rationale—including augmentation of cardiac output, reduction in filling pressures, and enhancement of contractility—have reliably produced favourable haemodynamic effects, yet have not altered the trajectory of disease. This disconnect reflects a multilevel mismatch in pathophysiology.

At the molecular and cellular levels, calcium flux amplification imposes substantial energetic and electrophysiological costs on an already compromised myocardium, exacerbating mitochondrial dysfunction and arrhythmogenic susceptibility. At the systemic level, improvements in macrocirculatory parameters do not necessarily restore effective tissue perfusion, as persistent abnormalities in microvascular flow, endothelial function, and cellular oxygen utilization may sustain organ dysfunction despite apparent hemodynamic stabilization. In addition, inotropic therapy is often introduced at advanced stages of disease, when structural injury, metabolic failure, and systemic inflammation may be only partially reversible.

These considerations suggest that the limitations of inotropic therapy are not simply pharmacological but reflect a fundamental discordance between the targets of intervention and the biology of advanced heart failure. Within this context, the potential relevance of approaches aimed at restoring, rather than amplifying, calcium cycling lies in their theoretical alignment with underlying pathophysiology; however, whether such strategies can overcome the historical dissociation between hemodynamic improvement and clinical outcomes remains an open question.

Despite a consistent and mechanistically plausible hemodynamic signal, the clinical relevance of istaroxime remains uncertain. The central limitation of inotropic therapy—demonstrated repeatedly across drug classes—is the dissociation between improvements in hemodynamic parameters and clinical outcomes.

**Figure 6:**
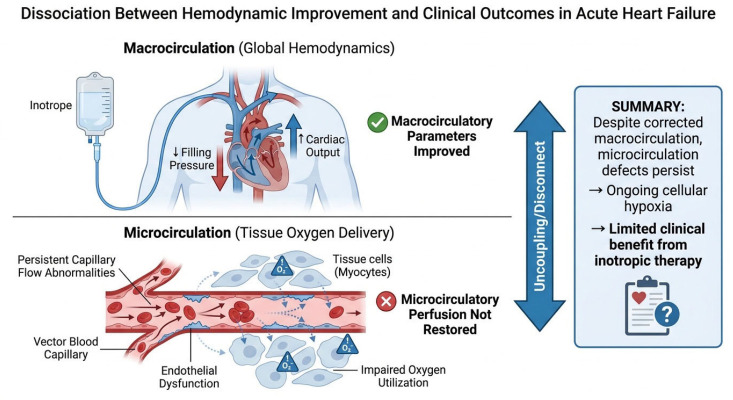
Dissociation between hemodynamic improvement and clinical outcomes in acute heart failure. Inotropic therapies consistently improve macrocirculatory parameters, including cardiac output and filling pressures. However, these changes do not necessarily translate into effective microcirculatory perfusion. Persistent abnormalities in capillary flow, endothelial function, and oxygen utilization may result in ongoing cellular hypoxia despite normalized global hemodynamics. This uncoupling between macrocirculatory improvement and microcirculatory perfusion provides a mechanistic explanation for the limited impact of inotropic therapy on clinical outcomes. ↑ increase; ↓ decrease. Created in FigureLabs; https://chat.figurelabs.ai/verify/FL-PUB-20260616-O0NDC2 (accessed on 16 June 2026).

A central limitation of inotropic therapy is the dissociation between macrocirculatory improvement and tissue-level perfusion [12,60,92] (Figure 6). While pharmacological agents reliably increase cardiac output and reduce filling pressures, these changes do not necessarily restore effective oxygen delivery at the microcirculatory level. Persistent alterations in capillary flow, endothelial function, and cellular oxygen utilization may result in ongoing tissue hypoxia despite apparent hemodynamic stabilization. This uncoupling provides a mechanistic explanation for the failure of inotropic therapies to translate physiological improvements into meaningful clinical benefit.

The current evidence base for istaroxime does not yet address this limitation. All available studies are phase II investigations [33,34,57] focused on physiological endpoints, with limited follow-up and no assessment of survival, organ recovery, or progression to advanced shock [93,94,95].

Furthermore, the relationship between macrocirculatory improvement and microcirculatory perfusion remains unresolved. In acute heart failure and cardiogenic shock, restoration of cardiac output does not necessarily translate into effective tissue oxygen delivery. Whether modulation of calcium cycling can favourably influence microvascular function or cellular energetics is unknown.

Finally, while early data suggest a favourable electrophysiological profile, the dual mechanism of istaroxime introduces complexities related to intracellular sodium handling that require further evaluation in larger and more diverse populations.

These considerations indicate that istaroxime should be viewed as a mechanistically innovative but still investigational therapy. Its ultimate role will depend not on its ability to improve haemodynamic surrogates, but on whether it can meaningfully alter the clinical trajectory of acute heart failure.

### 6.5. Unified Pathophysiological Model of Inotropic Failure and Calcium Cycling Restoration

The persistent failure of inotropic therapies to improve clinical outcomes despite consistent haemodynamic benefits suggests that their limitations are rooted in a multilevel pathophysiological mismatch rather than insufficient pharmacological potency. A unified framework integrating molecular, cellular, and systemic processes provides a coherent explanation for this dissociation.

At the molecular level, heart failure is characterized by dysregulation of calcium cycling rather than simple calcium deficiency. Impaired SERCA2a activity, persistent phospholamban-mediated inhibition, and pathological CaMKII activation result in inefficient calcium reuptake, diastolic cytosolic calcium accumulation, and reduced sarcoplasmic reticulum calcium stores. Concurrent RyR2 instability further promotes diastolic calcium leak, while alterations in sodium handling shift sodium–calcium exchanger activity, exacerbating intracellular calcium imbalance. These processes collectively produce a state of energetically inefficient and temporally disordered excitation–contraction coupling.

At the cellular and energetic level, these abnormalities impose a dual burden. Increased cytosolic calcium during diastole not only impairs relaxation but also drives ATP consumption through futile calcium cycling. Simultaneously, disrupted coupling between cytosolic and mitochondrial calcium handling limits activation of oxidative phosphorylation, reducing ATP availability. The result is a mismatch between energy demand and supply, in which attempts to augment contractility through calcium amplification further exacerbate metabolic stress.

At the organ and systemic level, this inefficiency contributes to the well-described dissociation between macrocirculatory improvement and microcirculatory perfusion. While conventional inotropes increase cardiac output and reduce filling pressures, they do not restore effective tissue oxygen delivery, in part due to persistent microvascular dysfunction, endothelial dysregulation, and impaired cellular oxygen utilization. This uncoupling explains why haemodynamic stabilization frequently fails to translate into improved organ function or survival.

Within this framework, conventional inotropes can be understood as agents that amplify an already dysregulated system. By increasing intracellular calcium flux without correcting underlying defects in calcium recirculation or energetics, they transiently improve systolic performance at the cost of increased metabolic demand and arrhythmogenic risk, reinforcing the fundamental pathophysiological imbalance.

Istaroxime represents a mechanistically distinct strategy that targets coordination rather than amplitude of calcium cycling. By simultaneously enhancing calcium availability and facilitating SERCA2a-mediated reuptake, it promotes restoration of temporal and energetic coupling within the cardiomyocyte. This integrated modulation has the potential to reduce diastolic calcium accumulation, improve contractile efficiency, and align energy utilization more closely with mechanical work.

However, whether restoration of calcium cycling at the molecular and cellular level is sufficient to overcome systemic constraints—particularly microcirculatory dysfunction and advanced metabolic failure—remains uncertain. The effectiveness of this approach is therefore likely to be phenotype-dependent, with greater relevance in early stages of haemodynamic compromise where cellular integrity is preserved and calcium cycling remains modifiable.

This unified model highlights that the central limitation of inotropic therapy lies not in the inability to increase cardiac output, but in the failure to restore coordinated function across molecular, cellular, and systemic domains. The therapeutic promise of istaroxime, and of calcium-cycling-targeted strategies more broadly, will ultimately depend on their capacity to bridge this multiscale gap.

## 7. Clinical Uncertainty and Translational Constraints of Istaroxime

Despite a mechanistically compelling profile and reproducible hemodynamic effects in early-phase studies, the clinical positioning of istaroxime remains defined more by promise than by proof. The current evidence base is largely restricted to phase II investigations powered for physiological endpoints rather than clinical outcomes and therefore cannot address the central question that has historically limited the adoption of inotropic therapies—whether improvement in cardiac performance translates into meaningful gains in survival, organ recovery, or durability of stabilization. This limitation is particularly relevant given the consistent dissociation observed with prior inotropes, where favorable hemodynamics failed to alter the trajectory of advanced heart failure.

Equally important is the phenotypic context of existing data. Patients enrolled in istaroxime studies have predominantly represented early hemodynamic compromise, often preceding overt cardiogenic shock. While this aligns with the biological premise that modulation of calcium cycling requires preserved cellular integrity, it also raises uncertainty about its applicability to more advanced stages of disease, where mitochondrial dysfunction, systemic inflammation, and microcirculatory failure predominate. In such settings, the extent to which restoration of intracellular calcium handling alone can meaningfully influence global hemodynamics remains uncertain.

From a mechanistic standpoint, the dual pharmacology of istaroxime—combining SERCA2a activation with Na^+^/K^+^-ATPase inhibition—raises important considerations. While the enhancement of calcium reuptake addresses a central defect in failing myocardium, the concomitant alteration in intracellular sodium handling introduces a layer of electrophysiological complexity that has not yet been fully characterized in larger or more heterogeneous populations. The absence of a strong proarrhythmic signal in early trials is reassuring but should be interpreted cautiously, given the limited exposure and controlled study conditions.

Tolerability, although generally acceptable, is not entirely neutral. Recurrent reports of gastrointestinal adverse effects [34,86] suggest off-target pharmacodynamic activity that, while not clinically severe, may influence real-world applicability in unstable patients. More broadly, the logistical and economic implications of introducing a novel intravenous inotrope into an already resource-intensive clinical setting remain undefined. Whether the haemodynamic profile of istaroxime translates into reduced escalation of care, shorter intensive care utilization, or improved cost-effectiveness is a critical, yet unanswered, question.

Despite its mechanistic appeal, istaroxime is unlikely to be appropriate across all clinical scenarios in acute heart failure. In advanced cardiogenic shock (SCAI stages C–E), where profound myocardial injury, systemic inflammation, and severe metabolic derangement predominate, modulation of intracellular calcium cycling alone is unlikely to restore adequate perfusion, and mechanical circulatory support remains the cornerstone of therapy [96,97].

Similarly, in patients with predominant right ventricular failure or significant pulmonary hypertension, agents with established pulmonary vasodilatory effects, such as phosphodiesterase-3 inhibitors, may be more effective, as istaroxime lacks a meaningful impact on pulmonary vascular resistance [52].

Its use may also be limited in patients with significant arrhythmic instability or pre-existing disturbances in sodium handling, where Na^+^/K^+^-ATPase inhibition could theoretically exacerbate electrophysiological vulnerability, despite the absence of a strong proarrhythmic signal in early studies.

In addition, in normotensive or hypertensive patients with preserved perfusion (warm–wet phenotype), where the primary therapeutic goal is afterload reduction and decongestion, vasodilatory strategies are more appropriate and the haemodynamic profile of istaroxime offers no clear advantage.

Finally, caution may be warranted in advanced stages of heart failure characterized by severe energetic depletion and mitochondrial dysfunction, where the capacity of SERCA2a-targeted interventions to meaningfully improve contractile performance may be limited. Collectively, these considerations suggest that istaroxime should be viewed as a phenotype-specific therapy with a potentially narrow therapeutic window, rather than a universal inotropic solution.

Taken together, these considerations highlight that istaroxime should currently be viewed not as a replacement for existing inotropes, but as a mechanistically innovative candidate whose clinical value remains to be established. Its ultimate role will depend on demonstrating that its favourable effects on calcium cycling and haemodynamic stability translate into outcomes that extend beyond surrogate improvement and meaningfully alter the course of acute heart failure.

### Comparison with Vasopressor–Inotrope Combinations

In clinical practice, inotropic therapy is frequently administered in combination with vasopressors rather than as isolated pharmacological interventions. The use of dobutamine or milrinone alongside norepinephrine reflects an attempt to balance opposing haemodynamic effects—augmentation of cardiac output with maintenance of arterial pressure. This strategy underscores a fundamental limitation of conventional inotropes: the need to pharmacologically counteract their intrinsic vasodilatory or hypotensive properties. Istaroxime, by contrast, demonstrates a haemodynamic profile characterized by simultaneous improvement in stroke volume and stabilization or increase in systolic blood pressure, raising the possibility that it may reduce reliance on combination therapy. However, this hypothesis remains untested. Whether istaroxime can replace or merely complement vasopressor–inotrope regimens will depend on its performance in more severe hemodynamic states and in direct comparative studies reflecting real-world practice.

## 8. Limitations of Current Evidence

Despite the promising mechanistic rationale and favorable hemodynamic profile observed in early clinical studies, the current evidence supporting istaroxime remains limited. Most available data originate from phase II clinical trials involving relatively small patient populations and short treatment durations. Consequently, the existing evidence base is primarily focused on surrogate hemodynamic and echocardiographic endpoints rather than clinically meaningful outcomes such as mortality, rehospitalization, progression of cardiogenic shock, recovery of end-organ function, or quality of life.

Another important limitation is the patient population studied to date. Most investigations have enrolled patients with acute heart failure accompanied by hypotension or early stages of cardiogenic shock, frequently corresponding to SCAI stage B physiology. Whether the haemodynamic benefits observed with istaroxime can be reproduced in patients with more advanced cardiogenic shock, severe systemic hypoperfusion, multiorgan dysfunction, or inflammatory shock states remains unknown.

The safety profile of istaroxime also requires further validation. Although no significant proarrhythmic signal has emerged in early studies, the number of treated patients remains relatively small and follow-up periods have been limited. Given the dual mechanisms of Na^+^/K^+^-ATPase inhibition and SERCA2a activation, the long-term electrophysiological consequences of therapy remain unclear.

In addition, several mechanistic hypotheses remain incompletely validated. Improved calcium cycling, enhanced lusitropy, and potential energetic advantages provide an attractive biological framework; however, whether these effects translate into measurable improvements in myocardial energetic efficiency, oxygen consumption, or long-term clinical outcomes remains to be established. Historical experience with other inotropic agents has repeatedly demonstrated that favorable physiological effects do not necessarily result in improved prognosis.

Despite promising haemodynamic and mechanistic findings, the current evidence base for istaroxime remains limited. Available data are derived primarily from small phase II studies focused on surrogate haemodynamic endpoints rather than clinically meaningful outcomes. Most enrolled patients had hypotensive acute heart failure or pre-cardiogenic shock physiology, with limited evidence in advanced cardiogenic shock. In addition, no randomized trial has directly compared istaroxime with established inotropes such as dobutamine, milrinone, or levosimendan using clinically meaningful outcome measures. Consequently, the effects of istaroxime on mortality, hospitalization, progression of shock, and long-term clinical outcomes remain uncertain.

Finally, no adequately powered head-to-head trials have compared istaroxime with established inotropes such as dobutamine, milrinone, or levosimendan. Therefore, the relative efficacy and safety of istaroxime within contemporary treatment algorithms remain uncertain. Large randomized trials evaluating hard clinical endpoints will be required before its precise role in the management of acute heart failure and cardiogenic shock can be defined.

## 9. Discussion

The development of inotropic therapy in acute heart failure has been marked less by progressive refinement than by a persistent failure to translate hemodynamic improvement into meaningful clinical benefit. Across multiple pharmacological classes, augmentation of cardiac output and reduction in filling pressures have been consistently achieved, yet these physiological gains have not altered the trajectory of the disease. This recurring dissociation suggests that the limitations of inotropic therapy are not merely pharmacological, but conceptual.

**Figure 7:**
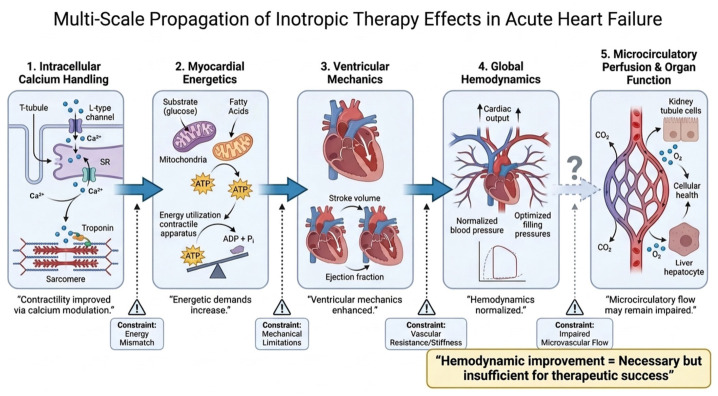
Multiscale failure of inotropic translation in acute heart failure. The effects of inotropic therapy originate at the level of intracellular calcium handling, where modulation of excitation–contraction coupling improves contractile performance. However, translation of this effect into clinical benefit requires propagation across multiple physiological scales, including myocardial energetics, ventricular mechanics, global hemodynamics, microcirculatory perfusion, and ultimately organ function. At each level, distinct constraints may attenuate or disrupt this transmission, resulting in progressive dissociation between cellular effects and clinical outcomes. This framework illustrates that improvement in hemodynamic parameters represents a necessary but insufficient condition for therapeutic success. Abbreviations: ADP, adenosine diphosphate; ATP, adenosine triphosphate; Ca^2+^, calcium ion; CO_2_, carbon dioxide; O_2_, oxygen; Pi, inorganic phosphate; SR, sarcoplasmic reticulum; T-tubule, transverse tubule. Created in FigureLabs; https://chat.figurelabs.ai/verify/FL-PUB-20260616-8ACMGA (accessed on 16 June 2026).

The limitations of inotropic therapy can be more fully understood within a multiscale framework that integrates cellular, organ-level, and systemic physiology (Figure 7). Pharmacological modulation of calcium handling primarily affects excitation–contraction coupling at the intracellular level, improving contractile performance and ventricular mechanics. However, translation of these effects into clinical benefit requires propagation across successive physiological domains, including myocardial energetics, global haemodynamics, and microcirculatory perfusion. At each level, additional constraints may attenuate or disrupt this transmission, resulting in progressive dissociation between improvements in cardiac output and effective tissue oxygen delivery. This multilevel uncoupling provides a unifying explanation for the persistent failure of inotropic therapies to improve outcomes and underscores that haemodynamic optimization, although necessary, is insufficient to ensure meaningful clinical benefit.

A systems-level perspective provides a unifying framework for understanding this limitation. The effects of inotropic therapy originate at the level of intracellular calcium handling, where modulation of excitation–contraction coupling improves contractile performance. However, translation of this effect into clinical benefit requires propagation across multiple physiological scales, including myocardial energetics, ventricular mechanics, global haemodynamics, microcirculatory perfusion, and ultimately organ function. At each level, additional constraints may attenuate or disrupt this transmission. As a result, improvement at the cellular or haemodynamic level does not necessarily translate into effective tissue oxygen delivery or improved clinical outcomes. This multiscale dissociation highlights that haemodynamic optimization represents a necessary but insufficient condition for therapeutic success and underscores the need for integrated strategies that address not only contractility, but also energetic efficiency, microvascular function, and systemic physiological coherence.

At a mechanistic level, conventional inotropes share a common strategy: the amplification of intracellular calcium flux [98,99]. While effective in increasing contractile force, this approach imposes a substantial energetic burden on a myocardium already characterized by mitochondrial dysfunction and impaired ATP generation. The result is a form of haemodynamic support that may be intrinsically unsustainable, as increased mechanical work is achieved at the expense of metabolic reserve. In parallel, the electrophysiological consequences of calcium overload—particularly diastolic calcium leak and triggered activity—are not secondary adverse effects but direct extensions of the underlying mechanism, contributing to the well-documented arrhythmogenicity of catecholaminergic therapies.

Equally important is the incomplete coupling between macrocirculatory improvement and tissue-level perfusion. In acute heart failure and cardiogenic shock, restoration of cardiac output does not necessarily translate into effective oxygen delivery or utilization. Microvascular dysfunction, endothelial activation, and capillary shunting may persist despite normalization of global haemodynamic parameters, resulting in a state in which organ hypoperfusion and cellular hypoxia remain unresolved. This limitation challenges the continued reliance on cardiac output as a surrogate endpoint and may partly explain the repeated failure of inotropic therapies to improve outcomes.

Within this context, istaroxime represents a mechanistically distinct approach that seeks to address a central abnormality of the failing myocardium—impaired calcium recirculation—rather than simply augment calcium availability. By combining Na^+^/K^+^-ATPase inhibition with enhancement of SERCA2a-mediated calcium reuptake, it promotes a more coordinated pattern of excitation–contraction coupling, integrating systolic augmentation with improved diastolic relaxation. This dual modulation results in a haemodynamic profile that differs in clinically relevant ways from conventional inotropes, characterized by increased stroke volume, preservation of heart rate, and stabilization or modest elevation of arterial pressure.

These properties are particularly relevant in the haemodynamic phenotype of hypotensive acute heart failure and early cardiogenic shock, where existing therapies often impose unfavourable trade-offs. β-adrenergic agonists provide rapid augmentation of cardiac output but at the cost of tachycardia and increased myocardial oxygen demand, while phosphodiesterase-3 inhibitors and calcium sensitizers frequently exacerbate hypotension through vasodilation. The ability of istaroxime to support both contractility and arterial pressure suggests that it may address a therapeutic niche not adequately covered by current pharmacological options.

However, the mechanistic coherence and favorable hemodynamic profile of istaroxime must be interpreted with appropriate caution. The history of inotropic therapy is replete with agents that demonstrated convincing physiological effects without translating into clinical benefit. The current evidence base for istaroxime is limited to early-phase studies evaluating hemodynamic endpoints and therefore does not address the central question of whether modulation of calcium cycling can meaningfully alter patient outcomes.

A phenotype-guided approach provides a physiologically coherent framework for the selection of inotropic therapy in acute heart failure. Rather than applying pharmacological agents uniformly, treatment may be aligned with the underlying hemodynamic profile, integrating cardiac output, congestion, vascular tone, and markers of end-organ perfusion. Within this framework, patients with low-output states (cold–wet and cold–dry phenotypes) represent the primary population in whom inotropic support is indicated, whereas individuals with preserved perfusion (warm–wet or warm–dry) may derive greater benefit from decongestive or vasodilatory strategies. Istaroxime, by combining augmentation of stroke volume with preservation of heart rate and stabilization of arterial pressure, exhibits a haemodynamic profile that may be particularly suited to hypotensive or early low-output states, in which conventional inotropes are limited by tachycardia or vasodilatory effects. However, this positioning remains hypothesis-generating and should be interpreted in the context of limited clinical evidence, as current data are derived predominantly from early-phase studies not designed to assess clinical outcomes. Accordingly, the proposed algorithm should be viewed as a conceptual model linking pathophysiology to therapeutic selection, rather than a definitive treatment recommendation.

Several additional uncertainties remain. The extent to which restoration of calcium recirculation improves myocardial energetics in vivo is not yet established, and the relationship between improved ventricular performance and microcirculatory function remains undefined. Moreover, the dual mechanism of istaroxime introduces electrophysiological considerations related to intracellular sodium handling that have not been fully characterized in larger or more heterogeneous populations. While early studies suggest a favourable safety profile, these observations require confirmation under real-world conditions.

Finally, the role of pharmacological inotropy must be considered within the evolving landscape of mechanical circulatory support. Temporary mechanical support devices provide direct haemodynamic augmentation and ventricular unloading without increasing myocardial oxygen demand, challenging the traditional reliance on inotropic agents in advanced cardiogenic shock. In this context, the potential value of istaroxime may lie not in replacing existing therapies, but in complementing them—either by stabilizing patients in early haemodynamic deterioration or by reducing exposure to high-dose catecholamines prior to escalation.

In conclusion, istaroxime represents a conceptual shift in the pharmacology of inotropic support, moving from indiscriminate calcium amplification to partial restoration of calcium cycling. Its hemodynamic profile suggests the possibility of more physiologically aligned support in selected patient populations. However, its clinical relevance will ultimately depend not on its ability to improve surrogate parameters, but on whether it can modify the natural history of acute heart failure—an objective that has thus far remained elusive across the entire class of inotropic therapies.

## 10. Future Directions in Acute Heart Failure Therapy: Beyond Conventional Inotropy

Future treatment of acute heart failure will likely shift away from a uniform hemodynamic approach toward earlier, phenotype-based interventions. Current ESC guidance already distinguishes between congestion-dominant presentations and hypoperfusion states, reserving inotropes for patients with systolic blood pressure < 90 mmHg and evidence of hypoperfusion despite standard therapy. This framework should evolve further toward rapid classification by congestion, perfusion, right- versus left-sided failure, vascular tone, renal reserve, lactate kinetics, and SCAI shock stage.

A major direction will be the development of agents that improve myocardial performance without simply increasing adrenergic drive or indiscriminate intracellular calcium flux. Istaroxime fits this concept because it targets calcium cycling more physiologically through SERCA2a activation while providing inotropic support through Na^+^/K^+^-ATPase inhibition. Future trials should therefore test whether this mechanism can reduce escalation to catecholamines, prevent progression from pre-shock to overt cardiogenic shock, shorten intensive care stay, and improve renal and hepatic recovery—not merely improve cardiac index or filling pressures.

Short-term mechanical circulatory support will also become increasingly central in AHF complicated by cardiogenic shock [100,101,102,103]. Devices such as intra-aortic balloon pump, Impella, left ventricular assist device, TandemHeart, and venoarterial extracorporeal membrane oxygenation should not be viewed simply as “last-resort rescue,” but as tools for early ventricular unloading, restoration of systemic perfusion, and reduction in toxic catecholamine exposure in selected patients. The key future question is not whether temporary MCS can raise blood pressure, but when, in whom, and with which unloading strategy it should be deployed. Contemporary shock frameworks emphasize that progression from SCAI stage B to C/D/E is dynamic, and delayed escalation may allow irreversible multiorgan injury.

The most promising therapeutic model may combine pharmacological and mechanical strategies. In early low-output AHF, an agent such as istaroxime could stabilize blood pressure, improve calcium cycling, and delay or prevent shock progression. In patients who deteriorate despite optimized pharmacology, early temporary MCS could provide circulatory support while minimizing prolonged exposure to catecholamines and high-dose conventional inotropes. Future studies should therefore evaluate integrated algorithms rather than isolated drugs or devices.

Finally, future AHF trials must move beyond short-term haemodynamic endpoints. Meaningful endpoints should include freedom from shock progression, lactate clearance, renal recovery, duration of ICU support, need for MCS, arrhythmia burden, rehospitalization, health–economic impact, and survival. This is particularly important for novel agents such as istaroxime, whose value will depend not only on haemodynamic superiority, but on whether improved myocardial efficiency translates into better clinical trajectories and lower resource utilization.

### Health Economics and Resource Utilization

The introduction of novel inotropic agents must be considered within the broader context of healthcare resource utilization. Acute heart failure and cardiogenic shock are among the most resource-intensive conditions in cardiovascular medicine, driven by prolonged intensive care stays, invasive monitoring, and frequent need for mechanical circulatory support.

While traditional inotropes are relatively inexpensive, their use is associated with complications that may prolong hospitalization and increase downstream costs. In contrast, newer agents such as istaroxime are likely to carry higher acquisition costs but may offer indirect economic benefits if they reduce escalation to advanced therapies, shorten ICU length of stay, or improve haemodynamic stability.

At present, no data exist to define the cost-effectiveness of istaroxime. Future studies should incorporate health–economic endpoints, as these will be critical in determining its adoption in clinical practice.

## 11. Conclusions

Inotropic therapy in acute heart failure has long been constrained by a fundamental paradox: the ability to improve haemodynamic parameters without altering the natural history of the disease. Across multiple drug classes, augmentation of contractility has been achieved primarily through amplification of intracellular calcium flux, a strategy that carries inherent energetic and electrophysiological costs and has consistently failed to translate into improved clinical outcomes.

Istaroxime represents a departure from this paradigm. By combining Na^+^/K^+^-ATPase inhibition with enhancement of SERCA2a-mediated calcium reuptake, it targets both systolic and diastolic dysfunction through modulation of calcium cycling rather than indiscriminate calcium amplification. This results in a haemodynamic profile characterized by increased stroke volume, preservation of heart rate, and stabilization of arterial pressure—features that distinguish it from conventional inotropes and suggest potential utility in hypotensive and early-stage haemodynamic compromise.

However, despite its mechanistic coherence and consistent haemodynamic effects, istaroxime remains an investigational therapy. The absence of outcome-driven evidence places it within the same evidentiary limitation that has historically defined inotropic pharmacology. Whether restoration of calcium cycling translates into improved myocardial efficiency, reduced arrhythmogenicity, and ultimately better clinical outcomes remains to be determined.

The future of inotropic therapy will depend not on further amplification of contractility, but on strategies that more effectively align myocardial performance with energetic capacity and systemic perfusion. Istaroxime represents one of the most conceptually advanced attempts to achieve this balance. Its ultimate role will be defined by its ability to move beyond hemodynamic improvement and meaningfully alter the clinical trajectory of acute heart failure.

## Figures and Tables

**Table 1:** Comparative Mechanistic Profile of Contemporary Inotropes.

Domain	Dobutamine [68]	Milrinone [69,70]	Levosimendan [22,23,71,72,73]	Istaroxime [32,34,61,75,76,77]
Primary mechanism	β1-adrenergic stimulation	PDE-3 inhibition	Troponin C sensitization	Na^+^/K^+^-ATPase inhibition + SERCA2a activation
Intracellular Ca^2+^	↑↑ (high flux)	↑↑ (high flux)	←→ (efficiency-based)	↑ (controlled, recirculated)
Calcium cycling	Amplification	Amplification	Utilization	Restoration of recirculation
Energetic demand	High	High	Moderate	Moderate (theoretical efficiency gain)
Heart rate	↑↑	←→/slight ↑	←→	←→
Blood pressure	Variable	↓	↓	↑/stabilized
Vascular effect	Mild vasodilation	Significant vasodilation	Pronounced vasodilation	Minimal vasodilation
Arrhythmogenicity	High	High	Intermediate	Potentially lower
Diastolic function	Limited	Moderate	Moderate	Pronounced improvement
β-blocker interaction	Reduced efficacy	Preserved	Preserved	Preserved
Clinical limitation	Tachycardia, ischemia	Hypotension	Hypotension, long action	Limited outcome data

Abbreviations: ATP—Adenosine triphosphate, β—Beta (adrenergic receptor), Ca^2+^—Calcium ion, Na^+^/K^+^-ATPase—Sodium–potassium adenosine triphosphatase, PDE-3—Phosphodiesterase type 3, SERCA2a—Sarcoplasmic reticulum Ca^2+^-ATPase isoform 2a. ↑ increase; ↑↑ marked increase; ↓ decrease; ←→ no overall change.

**Table 2:** Clinical Trials of Istaroxime.

Trial	Population	Haemodynamic Effects	Distinguishing Features	Clinical Implication	Reference
HORIZON-HF	AHF (reduced EF)	↑ CO, ↓ PCWP, improved diastolic function	No tachycardia, improved lusitropy	Proof of concept for dual inotropy + lusitropy	[32]
Istaroxime ADHF Trial	AHF (reduced EF)	Improved diastolic function	SBP increase, decrease in HR	Proof of concept for valid in AHF	[86]
SEISMiC	Hypotensive AHF (pre-shock)	↑ SBP, ↑ stroke volume, ↑ CO	BP increase without HR rise	Potential role in hypotensive patients	[34]
SEISMiC Extension	SCAI B shock	↑ CO and BP, stable HR	No arrhythmia signal	Early shock stabilization strategy	[87]
Overall Evidence	Phase II studies	Consistent haemodynamic improvement	Distinct from catecholamines	No outcome data; role remains investigational	---

Abbreviations: AHF—Acute heart failure, BP—Blood pressure, CO—Cardiac output, EF—left ventricular ejection fraction, HR—Heart rate, PCWP—Pulmonary capillary wedge pressure, SBP—Systolic blood pressure, SCAI—Society for Cardiovascular Angiography and Interventions. ↑ increase; ↓ decrease.

## Data Availability

No new data were created or analyzed in this study.

## Abbreviations

The following abbreviations are used in this manuscript:

AHF	Acute heart failure
AMP	Adenosine monophosphate
ATP	Adenosine triphosphate
ATPase	Adenosine triphosphatase
cAMP	Cyclic adenosine monophosphate
CS	Cardiogenic shock
DOREMI	Dobutamine Compared with Milrinone Trial
ESC	European Society of Cardiology
HF	Heart failure
HORIZON-HF	Hemodynamic Effects of Istaroxime in Patients with Heart Failure
ICU	Intensive care unit
K-ATP	ATP-sensitive potassium channel
MCS	Mechanical circulatory support
mmHg	Millimeters of mercury
NCX	Sodium–calcium exchanger
PDE-3	Phosphodiesterase type 3
PKA	Protein kinase A
REVIVE	Randomized Evaluation of Intravenous Levosimendan Efficacy
RV	Right ventricle/Right ventricular
SCAI	Society for Cardiovascular Angiography and Interventions
SEISMiC	Studies of Istaroxime in Cardiogenic Shock
SERCA2a	Sarcoplasmic reticulum Ca^2+^-ATPase isoform 2a
SURVIVE	Survival of Patients with Acute Heart Failure in Need of Intravenous Inotropic Support
